# Testing an early online intervention for the treatment of disturbed sleep during the COVID-19 pandemic (Sleep COVID-19): structured summary of a study protocol for a randomised controlled trial

**DOI:** 10.1186/s13063-020-04644-0

**Published:** 2020-08-08

**Authors:** Greg J. Elder, Pamela Alfonso-Miller, William C. M. Atkinson, Nayantara Santhi, Jason G. Ellis

**Affiliations:** Northumbria Sleep Research, Northumbria University, Newcastle upon Tyne, UK

**Keywords:** COVID-19, Randomised controlled trial, protocol, SARS-COV-2, acute insomnia, brief intervention, online intervention, CBT-I, short-term insomnia

## Abstract

**Objectives:**

The primary aim of the present study is to examine the efficacy of an online intervention for poor sleep in the context of an ongoing stressful major life event, by assessing if this intervention can reduce insomnia severity at short-term (one week post-intervention) and long-term (one and three months post-intervention) follow-up time points. It is hypothesised that the intervention will: 1) reduce insomnia severity in poor sleepers, compared to wait-list control poor sleepers, and good sleepers; 2) reduce subjective symptoms of anxiety and depression in all groups, and 3) prevent the transition to acute insomnia in good sleepers.

**Trial design:**

This study is a cluster randomised controlled trial.

**Participants:**

Both healthy good sleepers, who do not report having any current sleep problems, and individuals who report having sleep problems, will be recruited for the present study. This is a single-site study (Northumbria University). This study will be delivered using the internet and there are no geographic restrictions. Individuals who self-report as poor sleepers will meet DSM-5 criteria for acute insomnia, which is where individuals: 1) have difficulties in falling asleep, staying asleep, or awakening too early for at least three nights per week, for a time period of between two weeks and three months; and 2) report experiencing distress or impairment caused by sleep loss. Both 1) and 2) must have occurred despite the individual having had an adequate opportunity for sleep during this time period. Good sleepers will be individuals who do not have current sleep problems. All participants must have a sufficient level of English comprehension to understand and complete study measures. Individuals cannot participate if they report having chronic sleep problems (where they have existed for more than three months immediately prior to providing consent), nor will individuals who are actively seeking treatment for their sleep problems irrespective of how long they have had the sleep problem. Individuals also cannot participate if they have a self-reported history of head injuries, or if they have a self-reported diagnosis of schizophrenia, epilepsy or personality disorder, as the distraction techniques involved in the insomnia intervention may increase rumination in individuals with these conditions, and influence the effectiveness of the intervention.

**Intervention and comparator:**

Participants who receive the intervention will be provided with an online version of a self-help leaflet. A printed version of this leaflet has been successfully used in previous treatment studies, which have been conducted by our research group. Participants will be encouraged to download, save or print out this leaflet, which will be provided in PDF format. There will be no restrictions on use and participants will be encouraged to refer to this leaflet as often as they wish to. Briefly, this self-help leaflet aims to improve sleep by identifying and addressing sleep-related dysfunctional thinking by providing education about sleep, providing techniques to distract from intrusive worrisome thoughts at night, and providing guidelines for sleep-related stimulus control. The comparator is a wait-list control (i.e. where they will receive the intervention after a one month delay) group.

**Main outcomes:**

The primary outcome measure will be insomnia severity, as measured using the Insomnia Severity Index (Bastien, Vallières, & Morin, 2001), assessed immediately prior to the intervention and at one week, one month and three months post-intervention, compared to baseline. Secondary outcome measures will include subjective mood, measured using the 7-item Generalised Anxiety Disorder Questionnaire (GAD-7; Spitzer, Kroenke, Williams, & Lowe, 2006)) and 9-item Patient Health Questionnaire (PHQ-9; Kroenke, Spitzer, & Williams, 2001), assessed immediately prior to the intervention, and one week, one month and three months post-intervention, compared to baseline. Additionally, subjective sleep continuity, derived from sleep diaries (Carney *et al*., 2012), will be compared pre and post-intervention.

**Randomisation:**

This study will operate as a cluster randomised controlled trial. Good sleepers will be randomised into an intervention or a no-intervention group, with a 1:1 allocation. Poor sleepers will be randomised into an intervention or wait-list control group, with a 1:1 allocation. Randomisation will be conducted automatically using Qualtrics study software, where block sizes will be equal and randomisation will be computer-generated.

**Blinding (masking):**

Participants will not be blinded to group assignment. The outcomes will be assessed by a blinded investigator.

**Numbers to be randomised (sample size):**

The minimum sample size is 60. A total of 30 poor sleepers will be randomised to the intervention or wait-list control group. A total of 30 good sleepers will be randomised to the intervention or no intervention group.

**Trial Status:**

Recruitment for this study has yet to start. It is anticipated that recruitment will begin in August 2020 and end in April 2022. The current study protocol is version 1.0 (20 July 2020)

**Trial registration:**

This study was prospectively registered in the ISRCTN registry (registration number ISRCTN43900695, date of registration: 8 April 2020).

**Full protocol:**

The full protocol is attached as an additional file, accessible from the Trials website (Additional file 1). In the interest in expediting dissemination of this material, the familiar formatting has been eliminated; this Letter serves as a summary of the key elements of the full protocol.

The study protocol has been reported in accordance with the Standard Protocol Items: Recommendations for Clinical Interventional Trials (SPIRIT) guidelines (Additional file 2).

## Supplementary information

**Additional file 1.** Study protocol (v1.0, 20 July 2020).

**Additional file 2.** SPIRIT 2013 Checklist: Recommended items to address in a clinical trial protocol and related documents*.

## Data Availability

The research team will have the exclusive use of data for 12 months following the formal end of the trial. After this point, the final trial dataset will be available from the principal investigator, upon reasonable request by email (contact details are provided in the ISRCTN trial registration entry). Anonymised data would be provided. Data access will be provided in line with Northumbria University guidelines, however, data access will not reasonably be refused.

