# Supplementary material for: Testing an early online intervention for the treatment of disturbed sleep during the COVID-19 pandemic (Sleep COVID-19): structured summary of a study protocol for a randomised controlled trial
Source: Trials. 2020 Aug 8;21:704. doi: 10.1186/s13063-020-04644-0 (PMC7414282; doi:10.1186/s13063-020-04644-0)
Supplement: Supplementary file 1 — Additional file 1. Study protocol (v1.0, 20 July 2020). [file 13063_2020_4644_MOESM1_ESM.pdf]

Testing an early online intervention for the treatment of disturbed sleep during the COVID-19 pandemic (Sleep COVID-19)

Protocol Version: 1.0

Protocol Date: 20 July 2020

Funded by: Northumbria University

Sponsored by: Northumbria University

ISRCTN: ISRCTN43900695

Contents

1. WHO Trial Registration Data Set 4
2. Study contacts and roles 6
3. Author contributions: 6
4. Responsibilities 6
5. Funding 6
6. Sponsor details 6
7. Background 7
   7.1. Choice of comparators 8
   7.2. Study objectives 8
      7.2.1. Primary objective 8
      7.2.2. Secondary objectives 8
   7.3. Hypotheses 8
8. Methods 9
   8.1. Trial design 9
   8.2. Study setting 10
   8.3. Eligibility criteria 10
      8.3.1. Inclusion criteria 10
      8.3.2. Exclusion criteria 10
   8.4. Intervention 10
   8.5. Outcomes 11
      8.5.1. Primary Outcome Measures 11
      8.5.2. Secondary Outcome Measures 11
   8.6. Sample size 11
   8.7. Recruitment Strategy 12
   8.8. Measures 12
      8.8.1. Demographic and physical/psychiatric health information 12
      8.8.2. Sleep measures 12
      8.8.3. Psychological measures 13
      8.8.4. COVID-19 infection susceptibility and distress measures 13
      8.8.5. Sleep diaries and daily state-trait anxiety 13
   8.9. Procedure 13
      8.9.1. Baseline 15
      8.9.2. Sleep monitoring period 15
      8.9.3. Intervention 15
      8.9.4. Post-intervention 15

8.9.5. Follow-up periods. 16
8.10. Participant Timeline 16
8.11. Randomisation & Sequence Generation 18
8.12. Unblinding 18
9. Data Management 18
10. Statistical analysis 19
11. Adverse events 19
11.1. Definitions 19
11.2. Causality 20
11.3. Expected adverse reactions 21
12. Data monitoring and quality assurance 22
13. Modification of the protocol 22
14. Confidentiality 22
15. Declaration of Interests 23
16. Access to Data 23
17. Ancillary and post-trial care 23
18. Trial Results 23
19. Data availability and Data sharing 24
20. Authorship guidelines 24
21. References 25
22. APPENDIX 1: PARTICIPANT INFORMATION SHEET 28
23. APPENDIX 2: ONLINE CONSENT FORM 34
24. APPENDIX 3: INTERVENTION (SELF-HELP LEAFLET) 35
25. APPENDIX 4: PARTICIPANT DEBRIEF SHEET 37
26. APPENDIX 5: DEMOGRAPHIC AND HEALTH QUESTIONS 39
27. APPENDIX 6: EXAMPLE SOCIAL MEDIA ADVERTISEMENT 41

1. WHO Trial Registration Data Set

Data category	Information
Primary registry and trial identifying number	ISRCTN43900695
Date of registration in primary registry	8 April 2020
Secondary identifying numbers	Not applicable
Source(s) of monetary or material support	Northumbria University
Primary sponsor	Northumbria University
Secondary sponsor(s)	Not applicable
Contact for public queries	Dr. Greg Elder<br>Northumbria Sleep Research<br>Northumbria University<br>Newcastle upon Tyne, UK<br>NE1 8ST<br>g.elder@northumbria.ac.uk
Contact for scientific queries	Dr. Greg Elder: contact details as above
Public title	Testing an early online intervention for the treatment of disturbed sleep during the COVID-19 pandemic (Sleep COVID-19)
Scientific title	Testing an early online intervention for the treatment of disturbed sleep during the COVID-19 pandemic (Sleep COVID-19)
Countries of recruitment	Worldwide
Health condition(s) or problem(s) studied	Acute insomnia
Intervention	Online behavioural intervention
Key inclusion and exclusion criteria	*Ages eligible for study:* ≥18 years<br>*Sexes eligible for study:* both<br>*Accepts healthy volunteers: yes*<br>*Inclusion criteria:*<br>• Self-reported poor sleepers must meet DSM-5 criteria for acute insomnia.<br>• Participants must have an adequate level of English comprehension.<br>*Exclusion criteria:* Individuals with reported chronic sleep disturbances, and those who are currently seeking treatment for their insomnia, and individuals with a self- history of head injuries, a self-reported diagnosis of schizophrenia, epilepsy or personality disorder.
Study type	*Study type*: interventional<br>*Allocation*: randomised<br>*Intervention model:*<br>*Masking:* single-blind (outcomes assessor)<br>*Primary purpose:* treatment
Date of first enrolment	TBC (expected first enrolment August 2020)
Target sample size	60 (30 poor sleepers, 30 good sleepers)
Recruitment status	Not yet recruiting
Primary outcome(s)	Insomnia Severity Index scores (post-intervention)

Key secondary outcomes	• Subjective mood scores (PHQ-9 and GAD-7)<br>• Measures of sleep continuity derived from sleep diaries<br>• Insomnia Severity Index pre-post change scores
Study start date (date of initial ethical approval)	8 April 2020
Study end date	8 April 2022
Study duration	24 months

2. Study contacts and roles

Name	Role	Affiliation
Greg J Elder	Principal Investigator	Northumbria Sleep Research, Northumbria University
Pamela Alfonso-Miller	Co-Investigator	Northumbria Sleep Research, Northumbria University
Nayantara Santhi	Co-Investigator	Northumbria Sleep Research, Northumbria University
Jason G Ellis	Co-Investigator	Northumbria Sleep Research, Northumbria University

3. Author contributions:

GJE and JGE conceived and designed the study. PA-M and NS provided statistical expertise and assisted with the design of the study. All authors have approved the final protocol.

4. Responsibilities

GE will take lead responsibility for the design and conduct of the trial, the preparation of protocol and revisions, and for monitoring the overall progress of the study. NS will conduct the primary statistical analysis. GE and JE will take responsibility for the publication of study reports. PA-M will lead on recruitment. All named members will monitor study progress and will monitor data quality.

5. Funding

This study is financially supported by Northumbria University. This funding source has had no role in the design of this study and will not have any role during the execution of the study, in analyses and interpretation of data, or in decision to submit results.

6. Sponsor details

Organisation	Northumbria University
Contact name	Samantha King
Contact address:	Sutherland Building<br>Newcastle upon Tyne<br>NE1 8ST<br>United Kingdom
Telephone	0191 243 7108
Email:	samantha.king@northumbria.ac.uk

7. **Background**

Theoretical models of insomnia suggest that psychophysiological arousal, due to a stressful life event, can cause a short-term disruption to sleep (i.e. acute insomnia) (Spielman, Caruso, & Glovinsky, 1987; Spielman, Nunes, & Glovinsky, 1996). Over time, this can result in an individual engaging in maladaptive compensatory behaviours, such as spending excessive time in bed and becoming preoccupied with sleep, creating a long-term problem of poor sleep through behavioural conditioning (Ellis, Gehrman, Espie, Riemann, & Perlis, 2012).

Previous naturalistic studies have indicated that stressful events, in the form of natural disasters such as earthquakes or hurricanes, or events such as war, can disrupt sleep (Askenasy & Lewin, 1996; Kato, Asukai, Miyake, Minakawa, & Nishiyama, 1996; Mellman, David, Kulick-Bell, Hebding, & Nolan, 1995; Seelig et al., 2010). The ongoing COVID-19 pandemic may represent one such stressful life event. However, early interventions may present an opportunity to prevent a short-term sleep disruption from becoming a long-term clinical sleep problem; specifically from becoming chronic insomnia (Ellis, Cushing, & Germain, 2015).

One non-pharmacological treatment, which is highly effective in the treatment of chronic insomnia, is cognitive behavioural therapy for insomnia (CBT-I). CBT-I is a structured psychotherapy which has the aim of changing maladaptive cognition and behaviours which contribute to the development of insomnia (Randall, Nowakowski, & Ellis, 2019). CBT-I results in equivalent improvements to those observed using pharmacological (hypnotic) treatments, with more durable after-effects following treatment discontinuation, and with concomitant reductions in symptoms of anxiety and depression (Riemann et al., 2017). For these reasons, CBT-I is recommended as a first-line treatment for chronic insomnia (Riemann et al., 2017). However, CBT-I may be too time and resource-intensive to be feasible and practical in the treatment of acute insomnia, and shorter interventions are likely to be of benefit.

One previous study, which used a self-help pamphlet alongside a 60-70 minute single ("one shot") session of face-to-face CBT-I for acute insomnia, found that this method of treatment delivery was effective, on the basis of reductions on the Insomnia Severity Index (Ellis et al., 2015); follow-up studies have demonstrated efficacy when the treatment was delivered in a group format (Boullin, Ellwood, & Ellis, 2016) and in a male adult prison population (Randall et al., 2019). However, the use of the pamphlet alone, which is based on stimulus control, cognitive control, and imagery distraction techniques, will further increase the availability of brief interventions aimed at treating acute insomnia. Therefore, this intervention is well-suited to an online delivery model and can be used to reach a large number of people in the context of a large-scale stressful event. One potential limitation of the self-help pamphlet is that it is not yet known how long-lasting the effects of the intervention are. As such understanding this is necessary in order to maximise the efficacy of the intervention. Additionally, the use of such an intervention may aid the prevention of sleep problems in individuals with good sleep (i.e. where the intervention is used as a preventative tool).

Therefore, this study will examine if an online version of the self-help pamphlet is effective in reducing symptoms of acute insomnia in poor sleepers and determine if the effect is long-lasting, as well as investigate if the pamphlet is effective in the prevention of acute insomnia.

7.1. Choice of comparators

The intervention in this study is an online version of a self-help leaflet, which outlines the principles of stimulus control, cognitive control and imagery distraction techniques. This leaflet is framed as being "3D's": '*Detect*', which provides sleep diary instructions, '*Detach',* which provides stimulus control instructions, and '*Distract*', which refers to cognitive control and imagery distraction instructions.

This leaflet (Appendix 3) was originally developed to augment a single one-shot session of in-person CBT-I and has been successfully used in previous studies (Boullin et al., 2016; Ellis et al., 2015; Randall et al., 2019). Additionally, this leaflet, alone, has been shown to reduce cognitive and somatic arousal (Ellis et al., 2015).

Participants, who self-identify as poor sleepers in line with established criteria for acute insomnia, will receive the intervention. The comparator will be a wait-list control group of poor sleepers who will also receive the intervention, in addition to good sleepers who will receive the intervention, or who will not receive the intervention.

7.2. Study objectives

7.2.1. Primary objective

The primary aim of the present study is to examine the efficacy of an online intervention for poor sleep in the context of an ongoing stressful major life event, by assessing if this intervention can reduce insomnia severity at short-term (one week post-intervention) and long-term (one and three months post-intervention) follow-up.

7.2.2. Secondary objectives

The secondary aims of this study are to: assess if the intervention can improve subjective anxiety and depression in poor sleepers, assess if the intervention can improve sleep continuity (derived from subjective sleep diaries), assess the effectiveness of the intervention (on the basis of effect sizes), and examine if the intervention can prevent the transition to acute insomnia in good sleepers.

7.3. Hypotheses

It is hypothesised that the intervention will:

1) reduce insomnia severity in poor sleepers, compared to wait-list control poor sleepers, and good sleepers
2) reduce subjective symptoms of anxiety and depression in all groups.
3) prevent the transition to acute insomnia in good sleepers

8. **Methods**

8.1. Trial design

This study is designed as a cluster randomised controlled trial. Both self-reported good and poor sleepers will participate.

Good sleepers will be randomised into an intervention or a no intervention group, with a 1:1 allocation. Poor sleepers will be randomised into an intervention or wait-list control (i.e. where they will receive the intervention after a delay) group, with a 1:1 allocation. Overall, poor and good sleepers will participate at a 1:1 ratio (Figure 1).

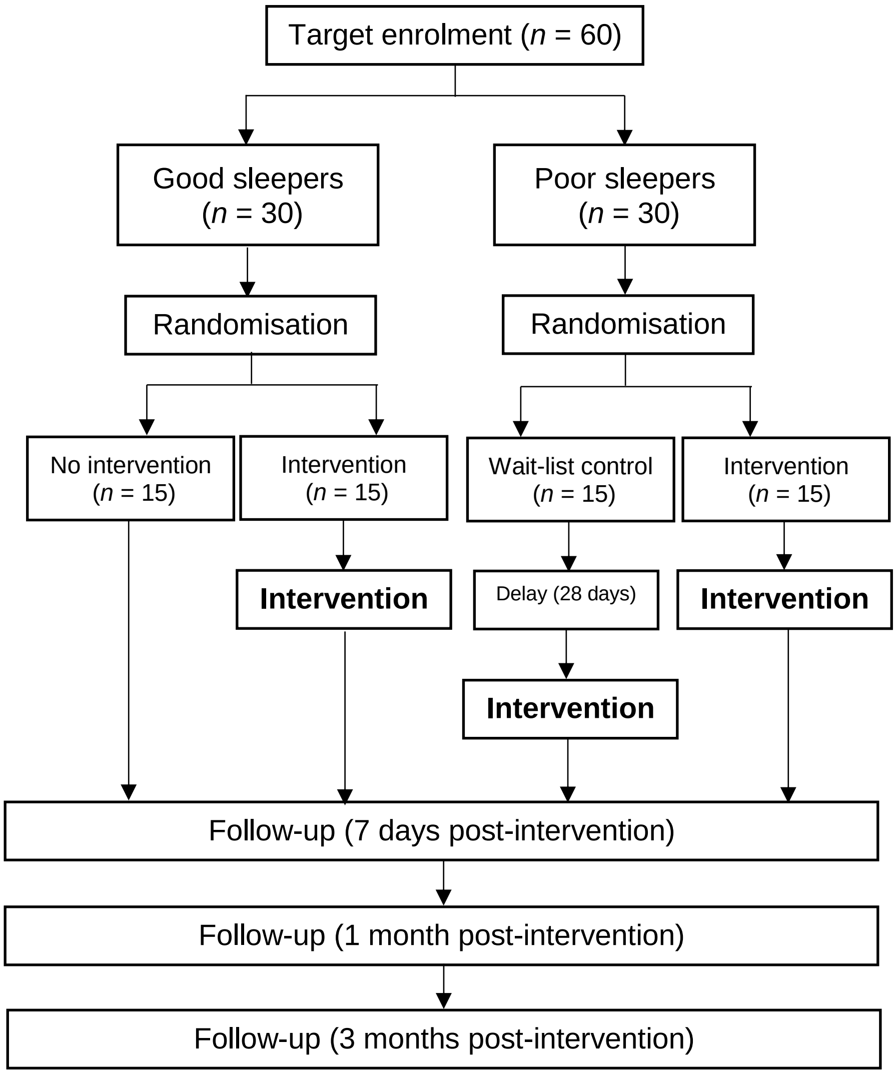

NB: good sleeper participants who do not receive the intervention will complete the follow-up stages at an equivalent time point.

Figure 1: participant flowchart

8.2. Study setting

This is a single-site study (Northumbria University). This study will be delivered using the internet and there are no geographic restrictions.

8.3. Eligibility criteria

Participants will be provided with an online information sheet (Appendix 1) and will be required to provide informed consent, which will be recorded electronically (Appendix 2), before any study procedures can be completed.

8.3.1. Inclusion criteria

Both healthy good sleepers, who do not report having any sleep problems, and individuals who do report current sleep problems (between two weeks and three months), will be recruited for the present study.

To participate, the following inclusion criteria will apply (to good and poor sleepers):

1) individuals must be aged 18 years or above
2) individuals must consider themselves to have a sufficient level of English comprehension to be able to understand and complete all study measures.

Self-reported poor sleepers will need to meet criteria for acute insomnia, as based on the definition included in the Diagnostic and Statistical Manual of Mental Disorders (DSM-5; American Psychiatric Association, 2013)

Specifically, individuals with poor sleep must report:

1) difficulties in falling asleep, staying asleep, or awakening too early for at least three nights per week, for a time period of between two weeks and three months
   AND
2) distress or impairment caused by sleep loss.

Both 1 and 2 must occur despite the individual having an adequate opportunity for sleep.

8.3.2. Exclusion criteria

Individuals who report having chronic sleep problems (i.e. where they have existed for more than three months immediately prior to providing consent) will not be eligible to participate nor will those who are actively seeking treatment for their sleep problems, irrespective of how long they have had the sleep problem. Additionally, participants who have a self-reported history of head injury, or who have had a diagnosis of schizophrenia, epilepsy or personality disorder cannot take part as the distraction techniques involved in the intervention may increase rumination and this may influence the effectiveness of the intervention.

8.4. Intervention

Participants who receive the intervention will be provided with an online version of a self-help leaflet (Appendix 3). A printed version of this leaflet has been successfully used in previous treatment studies, which have been conducted by our research group (Boullin et al., 2016;

Ellis et al., 2015; Randall et al., 2019). Participants will be encouraged to download, save or print out this leaflet, which will be provided in PDF format. There will be no restrictions on use and participants will be encouraged to refer to this as often as they wish to.

Briefly, this self-help leaflet aims to improve sleep by identifying and addressing sleep-related dysfunctional thinking by providing education about sleep, providing techniques to distract from intrusive worrisome thoughts at night, and providing guidelines for sleep-related stimulus control.

Treatment adherence will be formally monitored. This will be done by verifying that participants have downloaded, or have viewed, the information leaflet from the Qualtrics system. There are restrictions on pharmacological or non-pharmacological concomitant care before and during study participation as outlined in Section 8.3. This is necessary in order to be able to verify whether the intervention can reduce symptoms of acute insomnia.

8.5. Outcomes

8.5.1. Primary Outcome Measures

The primary outcome measures will be insomnia severity, as measured using the Insomnia Severity Index (Bastien, Vallières, & Morin, 2001), assessed prior to the intervention and one week, one month and three months post-intervention, relative to baseline.

8.5.2. Secondary Outcome Measures

The following measures are considered to be secondary outcome measures:

1) Subjective mood (subjective depression and anxiety), measured using the 7-item Generalised Anxiety Disorder Questionnaire (GAD-7) and 9-item Patient Health Questionnaire (PHQ-9). These will be assessed immediately prior to the intervention, one week, one month and three months post-intervention and will be compared to baseline.
2) Subjective sleep continuity (referring to the number of awakenings (NWAK), wake after sleep onset (WASO), total sleep time (TST), sleep onset latency (SOL) and sleep efficiency (SE%)), as derived from subjective sleep diaries (Carney et al., 2012), before and after the intervention.

8.6. Sample size

The sample size was calculated on the basis of the primary hypothesis.

An *a priori* power analysis, conducted using G*Power 3.1 (Faul, Erdfelder, Buchner, & Lang, 2009), indicated that a minimum of 48 participants are required on the basis of an expected medium effect size (f^2 = 0.25) at 95% power for the primary outcome measure (ISI). This was calculated using a 4 (group: poor sleeper / wait-list control / good sleeper intervention / good sleeper no intervention) x 5 (time point: baseline / pre-intervention / one week post-intervention / one month post-intervention / three months post-intervention) mixed analysis of variance (ANOVA) with an expected interaction.

It is anticipated that there will be an overall drop-out rate of 20% (n = 12) during the study and therefore a total sample size of 60 is required. An equal number of participants will be recruited to each group (n = 15). Poor and good sleepers will be recruited at a 1:1 ratio (30:30 participants).

8.7. Recruitment Strategy

The URL to the study will be placed online and this will be publicised to potential participants using the Northumbria University website and social media channels (e.g. the Northumbria University Twitter and Facebook account). The URL will also be made available to participants through the ISRCTN trial registration page.

There will not be any financial incentive for participation and the treatment will be offered free of charge to participants. As this is an intervention study, which is delivered online, we do not anticipate that there will be any difficulties with recruitment during the study period.

8.8. Measures

8.8.1. Demographic and physical/psychiatric health information

Participants will also be asked to provide a brief medical (physical and psychiatric) history and list of current medication, as well as occupational information (e.g. job title and role). Participants will also be asked questions which are related to COVID-19, including whether they have had a test for, a diagnosis of, or have shown symptoms of COVID-19, as well as whether or not they were furloughed (i.e. that they were placed on a temporary leave of absence from work by their employer), or were made unemployed during this period.

This information is required should a sufficient number of individuals participate; this will enable us to examine whether these variables impact on poor sleep, or the effectiveness of the intervention. For example, we will be able to assess whether individuals who are in particular job roles (e.g. healthcare) or with a significant number of existing health conditions, have poorer sleep quality in the context of the pandemic. To provide an indication of general health status, participants will complete the 36-Item Short Form Survey Instrument (SF-36; Ware & Sherbourne, 1992).

8.8.2. Sleep measures

Participants will complete the Sleep Disorders Symptom Checklist-25 (SDS-CL-25; Klingman, Jungquist, & Perlis, 2017), which indicates the presence or absence of common sleep disorders and will be used to verify that participants do not have any other sleep disorders, as well as subjective sleepiness (Karolinska Sleepiness Scale (KSS); Åkerstedt & Gillberg, 1990).

Participants will also complete the Mannheim Dream Questionnaire (MADRE; Schredl, 2014), which provides information regarding the frequency, content and emotional tone of dreams, and will be used to investigate whether these measures differ between good and poor sleepers, since individuals with insomnia have been shown to report more dreams of a negative nature than good sleepers.

Participants will also complete the Insomnia Severity Index (ISI; Bastien et al., 2001), which will be modified in order to assess insomnia severity during the preceding week, rather than assessing insomnia severity during the preceding month, as in the original measure.

8.8.3. Psychological measures

As general screening measures of subjective anxiety and depression, which are closely associated with insomnia, participants will complete the 7-item Generalised Anxiety Disorder Questionnaire (GAD-7; Spitzer, Kroenke, Williams, & Lowe, 2006) and 9-item Patient Health Questionnaire (PHQ-9; Kroenke, Spitzer, & Williams, 2001). The Perceived Stress Scale (PSS; Cohen, Kamarck, & Mermelstein, 1983), will be used to provide a subjective measure of stress throughout the study.

8.8.4. COVID-19 infection susceptibility and distress measures

In order to indicate how distressing the ongoing COVID-19 pandemic is considered to be, and to assess beliefs regarding infection susceptibility, and emotional discomfort in contexts which might imply an increased infection potential, participants will complete the Impact of Event Scale-Revised (Weiss, 2007) and Perceived Vulnerability to Disease questionnaire (Duncan, Schaller, & Park, 2009).

8.8.5. Sleep diaries and daily state-trait anxiety

Participants will complete the Consensus Sleep diary (CSD-M; Carney et al., 2012). This allows measures of sleep continuity (e.g. time in bed, total sleep time) to be derived, as well as measures of sleep quality. Participants will also complete the six-item version of the state scale of the Spielberger State-Trait Anxiety Inventory (STAI; Marteau & Bekker, 1992).

8.9. Procedure

The entire study will be conducted online, and will be delivered using Qualtrics. The full list of assessments to be completed at each time point is included in Table 1.

The end of study will be defined as the final follow-up time point (i.e. 3 months follow-up), or the final study measure completed, should the participant drop out of the study before this point.

Table 1: list of study assessments to be completed at each time point

**Measure**	**Baseline**	**Pre-intervention**	**Post-intervention**	**One week follow-up**	**1 Month follow-up**	**3 Month follow-up**
Demographic and physical/psychiatric health questions	X					
SDS-CL-25	X					
KSS	X	X	X	X	X	X
MADRE	X				X	X
GAD-7	X	X		X	X	X
PHQ-9	X	X		X	X	X
PSS	X	X		X	X	X
SF-36	X				X	X
Impact of Event Scale-R	X					
Perceived Vulnerability to Disease	X					
ISI	X	X		X	X	X
CSD-M		X	X			
STAI		X	X			

8.9.1. Baseline

After providing informed consent (online), good and poor sleeper participants will provide demographic information (e.g. age, sex), health and occupational information as described earlier. Participants will then complete the SDS-CL-25, KSS, MADRE, GAD-7, PHQ-9, PSS, SF-36, Impact of Event Scale-R, Perceived Vulnerability to Disease questionnaire and ISI. It is expected that the completion of baseline measures will take a maximum of 45 minutes.

Randomisation will also take place at this point. Good sleepers will be randomised to the intervention or no intervention group. Poor sleepers will be randomised to the intervention or wait-list control group.

8.9.2. Sleep monitoring period

Participants will then be e-mailed a link in order to complete a one-week period of sleep monitoring, which will be completed online. During this period, participants will complete subjective sleep diaries (CSD-M), subjective state-trait anxiety levels (STAI) and subjective sleepiness levels (KSS), approximately 30 minutes after awakening, by logging on to online survey software (Qualtrics) using a mobile phone or suitable internet-enabled device such as a laptop. It is expected that this will take participants approximately five to ten minutes per day to complete.

8.9.3. Intervention

Poor sleepers will receive the intervention after the one-week baseline period. Poor sleepers who are wait-list controls will receive the intervention after a delay of one month (28 days), and will extend their monitoring period accordingly, with extra ISI, GAD-7, PHQ-9 and PSS measures to be completed on Day 28 (Figure 2).

Participants will repeat the ISI, GAD-7, PHQ-9 and PSS immediately prior to receiving the intervention.

Good sleepers, who will receive the intervention, will do so at the same time as poor sleepers (Figure 2). Good sleepers who do not receive the intervention will follow an identical timeline to the poor sleepers who do receive the intervention (Figure 2).

Assessments will be modified at Day 8 (or the equivalent time point where appropriate) to only ask about the previous week.

8.9.4. Post-intervention

Following the intervention, all participants will complete daily CSD-M, KSS and STAI measures for one week. Good sleepers who do not receive the intervention will also complete daily CSD-M, KSS and STAI measures.

8.9.5. Follow-up periods

At one week follow-up (i.e. one week after the intervention), or an equivalent time point for good sleeper participants who do not receive the intervention, participants will complete the KSS, GAD-7, PHQ-9 and ISI. At one month and three months follow-up, participants will complete the KSS, GAD-7, PHQ-9, SF-36, MADRE and ISI. Participants will receive a debrief (Appendix 4) at the end of the study.

Assessments will be modified at follow-up points to only ask about the previous week, with the exception of the SF-36, which will not be modified.

8.10. Participant Timeline

A detailed participant timeline, with the end of study definition for each participant group, is included in Figure 2.

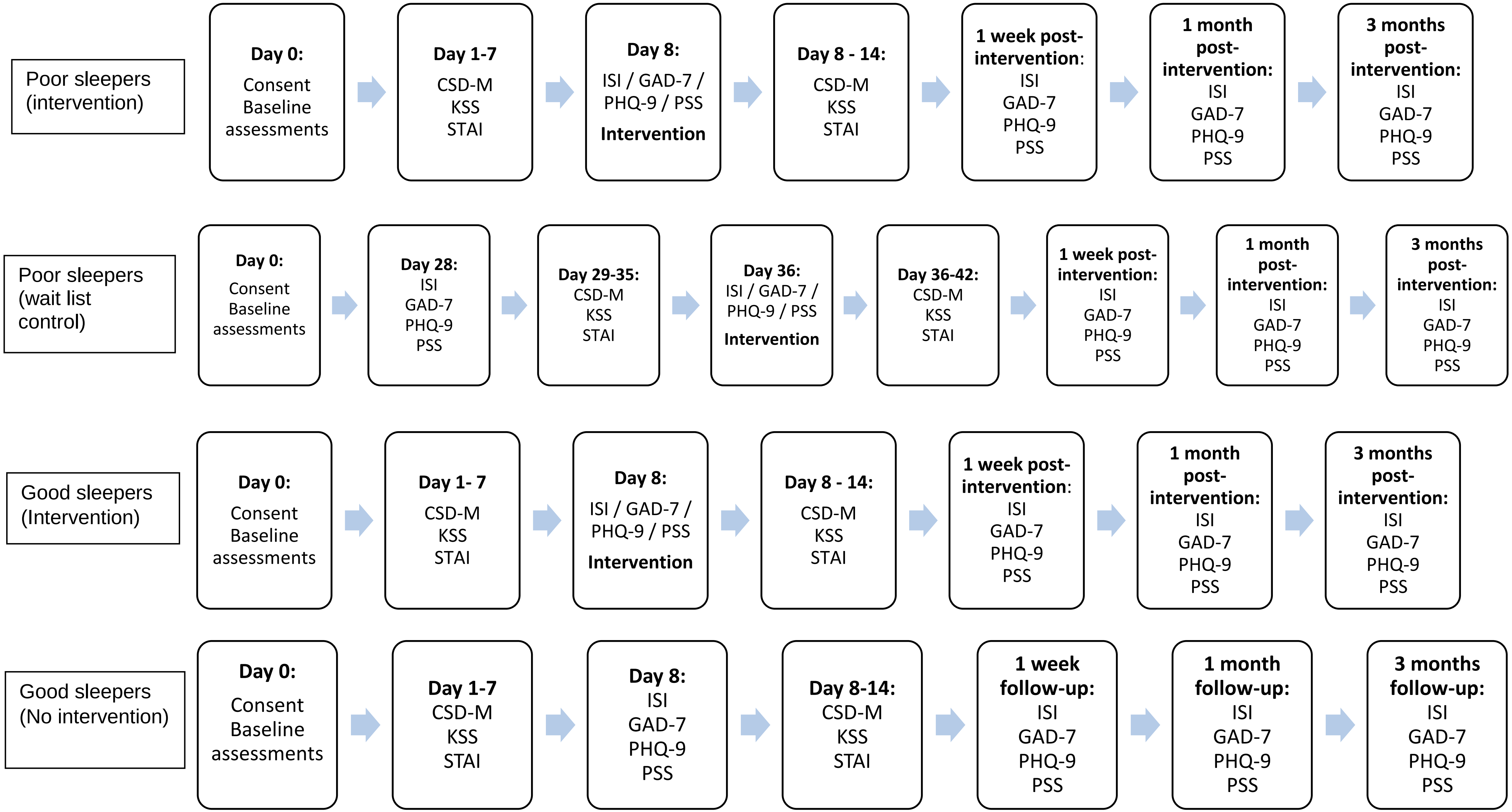

Figure 2: participant timeline

8.11. Randomisation & Sequence Generation

This study will operate as a cluster randomised controlled trial. Good sleepers will be randomised into an intervention or a no-intervention group, with a 1:1 allocation. Poor sleepers will be randomised into an intervention or wait-list control group, with a 1:1 allocation. Randomisation will be conducted automatically using Qualtrics study software, where block sizes will be equal and randomisation will be computer-generated.

Concealment will be assured, as Qualtrics will automatically allocate participants to the appropriate conditions after study entry, and will automatically direct participants to the appropriate online section of the study. Therefore, members of the study team will be unable to determine which arm good sleepers have been allocated to until after study entry. As the allocation sequence will be automatically generated by Qualtrics, randomisation will be conducted without any influence by any member of the study team.

All participants who give consent and fulfil the inclusion criteria will be randomised after the completion of baseline assessments. Due to the nature of the intervention, participants cannot be blinded to the study condition as they will be aware which condition they have been allocated to. For this reason, participants will be allocated a numeric group identification code in the study dataset to denote group allocation (1= good sleeper (no intervention); 2 = good sleeper (intervention); 3 = poor sleeper (wait-list control); 4 = poor sleeper. This is appropriate given the trial design.

The member of the research team who will be conducting the statistical analysis will be blinded to the study condition until the termination of the study. Any routine data monitoring will be undertaken by a member of the research team who will not be responsible for conducting the statistical analysis.

8.12. Unblinding

In order to maintain the overall quality of the trial, participant-level code breaks will only occur in exceptional circumstances, where the knowledge of the group is clinically essential for the management of the individual.

This risk of this situation is considered to be extremely low given the safety profile of the intervention and therefore it is not expected that this will be necessary during the trial. Where a code break is judged to be essential by the principal investigator, an independent member of the study team (i.e. who will not be undertaking any statistical analyses) will be permitted to unbreak the blinding by accessing the stored dataset.

If a code break occurs, the principal investigator will maintain the blind as much is possible. Whilst the allocation will be known to the participant, in all circumstances, the allocation is not to be disclosed to any other study personnel or the sponsor. The written or verbal disclosure of the code is only to be done where this is clinically necessary. The principal investigator is required to report all code breaks, with the reason for the code break, on an electronic case report file (CRF) page and this must be documented.

9. Data Management

All study data will be electronic and will be obtained from data directly input from the participant. Participant informed consent will be provided electronically. All CRFs and

documentation will be electronic. Paper copies of data or forms will not be held unless they are required in exceptional circumstances (e.g. if CRFs are required by the sponsor); paper copies of documentation will be retained securely in line with standard Northumbria University guidelines.

The online component of the study, and resulting dataset, will be extensively tested prior to the first participant enrolment, to ensure that all methods of data entry are reliable.

The full dataset will be checked and verified for integrity at the end of the study by the principal investigator, and/or other members of the study team. The dataset will also be checked to verify the integrity and quality of data at regular intervals. Any necessary changes to the dataset will be fully documented using a written log.

Anonymised data, and relevant documentation/CRFs will be stored on secure, password-protected computer storage accessible only by the research team. Data will be stored in line with standard university retention guidelines, and will be backed up regularly by Northumbria University IT Services.

10. **Statistical analysis**

Statistical analysis will be conducted by a member of the team who will not have any role in checking the data during the study (NS). Due to the small target sample size, interim analyses will not be conducted. Analysis will be conducted either at the end of the study duration, or when the recruitment target is met.

Data will be analysed using an intention-to-treat approach. The primary outcome analysis will be based only on the complete case/observed outcomes and imputation of missing data will not take place, since there is no reason to believe that participants who may be lost to follow-up will occur randomly. Summary information (i.e. descriptive statistics) will be calculated and presented as mean values (with standard deviations) or percentages, as required.

The primary outcome measure (ISI) will be analysed using a 4 (group: poor sleeper / poor sleeper wait-list control / good sleeper intervention / good sleeper no intervention) x 5 (time point: baseline / pre-intervention / one week post-intervention / one month post-intervention / three months post-intervention) mixed analysis of variance (ANOVA). A significant interaction will be followed up using *post hoc* tests. Effect sizes (partial eta squared) will be derived from ANOVAs and will be used to demonstrate effectiveness

Secondary outcome measures will be assessed using 4 (group) x 5 (time point) ANOVAs, or 4 (group) x 2 (pre-intervention and post-intervention) ANOVAs in the case of sleep diaries, adjusted for multiple comparisons where appropriate (in the case of sleep continuity)

Remission rates will be calculated on the basis of a score of 10 or more on the ISI at follow-up and as a percentage of groups. In the good sleeper participants, those who transition to subsequently having insomnia will be defined as those individual who display a score of <10 points on the ISI at baseline, and >10 points on the ISI at follow-up stages.

11. **Adverse events**

11.1. Definitions

Adverse event (AE): any untoward medical occurrence in a participant to whom a study intervention has been administered, including occurrences which are not necessarily caused

by, or related to, that intervention. An AE, therefore, does not necessarily have a causal relationship with the intervention. Medical conditions or diseases which are present before starting the study are only considered adverse events if they worsen after the intervention.

Related AE: an AE that results from administration from any of the research study procedures. All AEs judged by the principal investigator, or the sponsor, as having a reasonable causal relationship to a study procedure qualify as a related AE. The expression “reasonable causal relationship” means to convey in general that there is evidence or argument to suggest a causal relationship.

11.2. Causality

The assignment of the causality will be made by the principal investigator using the definitions in the table below. All AEs judged as having a reasonable suspected causal relationship to a study procedure (i.e. definitely, probably or possibly related) are considered to be related AEs. In the case of discrepant views between the principal investigators and others, all parties will discuss the case. In the event that no agreement is made, the principal investigator will make the final decision and both points of view will be documented.

Table 3: explanation of adverse event causality

**Relationship**	**Description**
Unrelated	There is no evidence of any causal relationship
Unlikely	There is little evidence to suggest there is a causal relationship (e.g. the event did not occur within a reasonable time after administration of the study procedure). There is another reasonable explanation for the event (e.g. the participant’s clinical condition, other concomitant treatment).
Possible	There is some evidence to suggest a causal relationship (e.g. because the event occurs within a reasonable time after administration of the study procedure). However, the influence of other factors may have contributed to the event (e.g. the participant’s clinical condition, other concomitant treatments).
Probable	There is evidence to suggest a causal relationship and the influence of other factors is unlikely.
Definitely	There is clear evidence to suggest a causal relationship and other possible contributing factors can be ruled out.
Not assessable	There is insufficient or incomplete evidence to make a clinical judgement of the causal relationship.

Unexpected Adverse Event: An AE that is not listed in the study protocol as an expected occurrence in the circumstances of this trial.

Serious Adverse Event (SAE): an untoward occurrence, whether expected or not, that results in death; is life-threatening (referring to an event in which the participant was at risk of death, not an event which hypothetically might have caused death if it were more severe); requires hospitalisation, or prolongation of existing hospitalisation; results in persistent or significant disability or incapacity; is otherwise considered medically significant by the principal investigator. Important medical events that are not immediately life-threatening, or do not result in death or hospitalisation, but that may jeopardise the individual or require

intervention to prevent one of the other outcomes listed above, should also be considered serious.

The severity of all AEs will be graded on a three-point scale of intensity (mild, moderate, severe):

- *Mild:* discomfort is noticed but there is no disruption of normal daily activities
- *Moderate:* discomfort is sufficient to reduce or affect normal daily activities
- *Severe*: discomfort is incapacitating, with an inability to work or to perform normal daily activities. An AE may be severe but not serious.

11.3. Expected adverse reactions

Expected AEs are summarised below. All AEs are expected to be common and well-understood consequences of study participation.

**Procedure**	**Adverse event**
Study questionnaires	Low levels of psychological discomfort
Study sleep intervention	Heightened sensitivity to poor sleep

As the intervention will be self-administered, participants will be entirely free to discontinue treatment.

For the purposes of this study, any AEs which the investigators are notified of during the study (i.e. from Day 0 to the final study measure) will be recorded. All AEs will be categorised as to expectedness, relatedness and severity.

Serious AEs exclude any pre-planned hospitalisations (e.g. elective surgery) not associated with clinical deterioration; routine treatment or monitoring of the studied indication which is not associated with clinical deterioration; elective or scheduled treatment for pre-existing conditions that did not worsen during the study; serious AEs exclude changes in insomnia severity or sleep, which are already documented within the study.

All AEs will be reported. Any questions concerning AE reporting will be directed to the principal investigator.

AEs: all non-serious AEs will be reported using electronic CRFs and sent to the principal investigator as soon as possible. The severity of AEs will be graded on a three-point scale (mild, moderate or severe). The relation (causality) and seriousness of the AE will be assessed by the principal investigator.

SAEs: all SAEs will be reported to the principal investigator within 24 hours of the research team learning of its occurrence. The initial SAE report can be made by telephone or e-mail to the principal investigator. In the case of incomplete information at the time of initial reporting, all appropriate information should be provided as follow-up as soon as this becomes available. Relationship of the SAE to study procedures will be assessed by the principal investigator, as should the expected or unexpected nature of the AE. The sponsor does not need to be informed of SAEs.

Evidence of SAEs will trigger a study monitoring meeting, involving the entire research team, to clarify if the trial will need to be discontinued on the basis of these events. The research

team will have access to all data accrued to that point and will have the power to terminate the trial early.

12. Data monitoring and quality assurance

As this is a study with an extremely low risk of side effects due to the behavioural nature of the intervention, a formal Trial Steering Committee, or Data Monitoring Committee, is not required for this study. The trial will be managed and monitored locally by a study management group consisting of the named investigators. Quality control will be maintained through adherence to all relevant Northumbria University standard operating and research governance procedures and the principles of Good Clinical Practice. However, the investigators will permit the sponsor to conduct study-related monitoring, audits and any other inspection, and will provide direct access to source data and study documentation where this is required and requested.

A brief version of this study protocol has already been reviewed by the Northumbria University Department of Psychology ethics committee and ethical approval has been granted (Reference 23377, date of approval: 6 April 2020). All study measures and participant documentation (information sheets, consent forms and debrief forms) have been reviewed and approved by two independent reviewers.

Whilst it is not required, in the interests of transparency, the Northumbria University research ethics committee will be supplied with a copy of the full protocol for reference.

13. Modification of the protocol

Any modifications to the protocol which may impact upon the conduct of the study (e.g. objectives, design, population, sample sizes, study procedures) will require a formal amendment to the protocol and ethical amendment. These will be formally documented on the most recent version of the study protocol, where a summary of changes will be provided and documented.

14. Confidentiality

All study data will be electronic, and paper copies of data will not be held. All personal data will be regarded as being strictly confidential. The study will comply with the requirements of the General Data Protection Regulation (GDPR). Participant email addresses will be collected with the sole purpose of sending participants reminders to complete their daily sleep diary, and appropriate follow-up stages of the study. Email addresses will not be collected for any other purpose and will be deleted upon study completion.

Anonymised data will be stored on secure, password-protected computer storage that will be accessible only by members of the research team who have the appropriate login credentials. Access may be delegated to other members of staff who have the appropriate authorisation; their access will only be provided to the anonymised dataset at the end of the study.

All electronic data will be stored in line with standard Northumbria University retention guidelines and in accordance with all other relevant legislation (e.g. GDPR). All members of the research team will have appropriate GDPR training.

In order to maximise the scientific value of the dataset, we intend to combine anonymised data from this study with data from similar studies conducted within this research group, and where possible, larger collaborative studies. Provision has been made in the participant consent form to allow for this and participants are permitted to opt out of this if they wish to do so.

15. **Declaration of Interests**

No members of the study team have any competing interests to declare.

16. **Access to Data**

This is a single site study, where the intervention and all study procedures will be delivered online. For that reason, no other external partners are involved and the transfer of data is not required.

GJE and JGE will have access to the full dataset as described in the relevant section. NS will have access to the full dataset at the end of the study in order to conduct all statistical analyses.

17. **Ancillary and post-trial care**

Due to the minimal risk of side effects, there is no provision for ancillary care.

As the nature of the intervention is focussed on acute (short-term) insomnia, the provision of this treatment after the study is not necessary, as the intervention is not designed for chronic insomnia and its efficacy is unknown. However, participants will be reminded that they should contact their GP should they have any further sleep problems. Should any participants in the good sleeper no-intervention group (i.e. those who do not receive the intervention) report having developed short-term sleep problems during the study, the online intervention will subsequently be offered to them, free of charge.

Northumbria University has insurance to cover non-negligent harm associated with the protocol.

This study is very unlikely to cause any psychological distress. The intervention is a safe and established treatment and it has been successfully used in previous studies within our group and we do not expect there to be any resulting side effects. However, we will direct participants who are concerned about the psychological impact of study participation to their GP. If participants are concerned about their physical health, they will be referred primarily to the NHS 111 website (for participants who are in the UK) and also to their GP.

18. **Trial Results**

It is anticipated that the results of the project will be published in peer-reviewed journals and will be reported at relevant national and international scientific meetings. It is anticipated that a peer-reviewed publication, with the aim of addressing the primary hypothesis, will be submitted to an appropriate journal within 12 months of the formal end date of the study.

Participants will be able to request a copy of the study results upon completion of the study. Additionally, we will aim to provide a summary of findings on the Northumbria University

website. We will provide ISRCTN with appropriate links to publications summarising the study results.

The results of study may also be reported to the sponsor, where appropriate. No individuals will be identified from any study report.

19. Data availability and Data sharing

The data will be the property of the Principal Investigator who will also be responsible for subsequent publication of the results.

The datasets generated during and/or analysed during the current study will be available from the principal investigator upon reasonable request (after a period of exclusive use (12 months). Our intended policy is that the research team will have exclusive use of the data for a period of 12 months from the end of the project, or until the data is published, if this is required alongside publications. Anonymised data will be provided and data will be made available upon application and the research team would control access in line with Northumbria University guidelines, however, data access will not reasonably be refused. There is no provision for public access to the protocol, statistical code and dataset, although reasonable requests will be accommodated in line with the intended use policy stated above.

20. Authorship guidelines

The named members of the research team will be named as co-authors on all related manuscript and conference submissions, where they have made substantive contributions to the design, conduct, interpretation, statistical analysis and reporting of the trial. Lead authorship will be negotiated on a case-by-case basis with the research team. Additional authors may be permitted, with the agreement of the research team, in situations where the additional authors have made sufficient contributions to merit authorship. The research team do not intend to use any external professional writers in order to generate any study publications.

21. References

Åkerstedt, T., & Gillberg, M. (1990). Subjective and objective sleepiness in the active individual. *International Journal of Neuroscience, 52*(1-2), 29-37.

American Psychiatric Association. (2013). *Diagnostic and Statistical Manual of Mental Disorders : DSM-5* (5th ed.). Washington, DC: American Psychiatric Association.

Askenasy, J. J., & Lewin, I. (1996). The impact of missile warfare on self-reported sleep quality. Part 1. *Sleep, 19*(1), 47-51.

Bastien, C. H., Vallières, A., & Morin, C. M. (2001). Validation of the Insomnia Severity Index as an outcome measure for insomnia research. *Sleep Medicine, 2*(4), 297-307.

Boullin, P., Ellwood, C., & Ellis, J. G. (2016). Group vs. Individual Treatment for Acute Insomnia: A Pilot Study Evaluating a "One-Shot" Treatment Strategy. *Brain Sci, 7*(1).

Carney, C. E., Buysse, D. J., Ancoli-Israel, S., Edinger, J. D., Krystal, A. D., Lichstein, K. L., & Morin, C. M. (2012). The consensus sleep diary: standardizing prospective sleep self-monitoring. *Sleep, 35*(2), 287-302.

Cohen, S., Kamarck, T., & Mermelstein, R. (1983). A global measure of perceived stress. *Journal of Health and Social Behavior, 24*(4), 385-396.

Duncan, L. A., Schaller, M., & Park, J. H. (2009). Perceived vulnerability to disease: Development and validation of a 15-item self-report instrument. *Personality and Individual Differences, 47*(6), 541-546.

Ellis, J. G., Cushing, T., & Germain, A. (2015). Treating Acute Insomnia: A Randomized Controlled Trial of a "Single-Shot" of Cognitive Behavioral Therapy for Insomnia. *Sleep, 38*(6), 971-978.

Ellis, J. G., Gehrman, P., Espie, C. A., Riemann, D., & Perlis, M. L. (2012). Acute insomnia: Current conceptualizations and future directions. *Sleep Medicine Reviews, 16*(1), 5 - 14.

Faul, F., Erdfelder, E., Buchner, A., & Lang, A. G. (2009). Statistical power analyses using G*Power 3.1: tests for correlation and regression analyses. *Behavior Research Methods, 41*(4), 1149-1160.

Kato, H., Asukai, N., Miyake, Y., Minakawa, K., & Nishiyama, A. (1996). Post-traumatic symptoms among younger and elderly evacuees in the early stages following the 1995 Hanshin-Awaji earthquake in Japan. *Acta Psychiatrica Scandinavica, 93*(6), 477-481.

Klingman, K. J., Jungquist, C. R., & Perlis, M. L. (2017). Introducing the Sleep Disorders Symptom Checklist-25: A Primary Care Friendly and Comprehensive Screener for Sleep Disorders. *Sleep Med Res, 8*(1), 17-25.

Kroenke, K., Spitzer, R. L., & Williams, J. B. (2001). The PHQ-9: validity of a brief depression severity measure. *Journal of General Internal Medicine, 16*(9), 606-613.

Marteau, T. M., & Bekker, H. (1992). The development of a six-item short-form of the state scale of the Spielberger State-Trait Anxiety Inventory (STAI). *British Journal of Clinical Psychology, 31*(3), 301-306.

Mellman, T. A., David, D., Kulick-Bell, R., Hebding, J., & Nolan, B. (1995). Sleep disturbance and its relationship to psychiatric morbidity after Hurricane Andrew. *The American Journal of Psychiatry, 152*(11), 1659-1663.

Randall, C., Nowakowski, S., & Ellis, J. G. (2019). Managing Acute Insomnia in Prison: Evaluation of a "One-Shot" Cognitive Behavioral Therapy for Insomnia (CBT-I) Intervention. *Behavioral Sleep Medicine, 17*(6), 827-836.

Riemann, D., Baglioni, C., Bassetti, C., Bjorvatn, B., Dolenc Groselj, L., Ellis, J. G., . . . Spiegelhalder, K. (2017). European guideline for the diagnosis and treatment of insomnia. *Journal of Sleep Research, 26*(6), 675-700.

Schredl, M. (2014). Appendix: The Mannheim Dream Questionnaire (MADRE). *International Journal of Dream Research, 7*(2).

Seelig, A. D., Jacobson, I. G., Smith, B., Hooper, T. I., Boyko, E. J., Gackstetter, G. D., . . . Millennium Cohort Study, T. (2010). Sleep patterns before, during, and after deployment to Iraq and Afghanistan. *Sleep, 33*(12), 1615-1622.

Spielman, A. J., Caruso, L. S., & Glovinsky, P. B. (1987). A behavioral perspective on insomnia treatment. *The Psychiatric Clinics of North America, 10*(4), 541-553.

Spielman, A. J., Nunes, J., & Glovinsky, P. B. (1996). Insomnia. *Neurologic Clinics, 14*(3), 513-543.

Spitzer, R. L., Kroenke, K., Williams, J. B., & Lowe, B. (2006). A brief measure for assessing generalized anxiety disorder: the GAD-7. *Archives of Internal Medicine, 166*(10), 1092-1097.

Ware, J. E., Jr., & Sherbourne, C. D. (1992). The MOS 36-item short-form health survey (SF-36). I. Conceptual framework and item selection. *Medical Care, 30*(6), 473-483.

Weiss, D. S. (2007). The Impact of Event Scale: Revised. In J. P. Wilson & C. S.-k. Tang (Eds.), *Cross-Cultural Assessment of Psychological Trauma and PTSD* (pp. 219-238). Boston, MA: Springer US.

22. APPENDIX 1: PARTICIPANT INFORMATION SHEET

Northumbria University NEWCASTLE

Faculty of Health & Life Sciences

Study title: Testing an early online intervention for the treatment of disturbed sleep during the COVID-19 pandemic (Sleep COVID-19)

Participant Information Sheet

You are being invited to take part in this research study. Before you decide it is important for you to read this leaflet so you understand why the study is being carried out and what it will involve.

Reading this leaflet, discussing it with others or asking any questions you might have will help you decide whether or not you would like to take part.

What is the Purpose of the Study?

Stressful life events can cause a short-term disruption to sleep. This can cause people to try and compensate for the sleep disruption. For example, people might then spend too long in bed, or become preoccupied with the daytime consequences of this poor or disturbed sleep. Over time, we know that this can create long-term sleep problem such as insomnia.

Previous research studies have shown that stressful major events, in the form of natural disasters such as earthquakes, can disrupt sleep. The ongoing COVID-19 (coronavirus) pandemic might therefore cause people to develop sleep problems. However, by intervening early, we think that short-term sleep disruption can be stopped. This is likely to prevent short-term sleep disruption from becoming a long-term problem.

The aim of this study is to use an online treatment, in the form of sleep education, to try and treat short-term sleep problems in people who have recently reported having poor sleep, particularly as a result of the ongoing COVID-19 pandemic. This involves being provided with an online version of a leaflet which suggests ways in which people can change their behaviour to avoid poor sleep from becoming a problem. We have previously used a version of this leaflet with people who have long-term sleep problems (insomnia) and it has been effective. We want to know if this is effective in the short-term (one week after receiving the leaflet) and longer-term (one and three months later)

We are also looking for people who do not have sleep problems (good sleepers) to take part in this study because we want to understand if this intervention can prevent sleep problems from happening in the first place. We also want to track the sleep of good sleepers who do not have any sleep problems, and who do not receive the intervention, to see what happens to sleep under normal circumstances.
This study has been registered as a clinical trial on the ISRCTN database (**https://www.isrctn.com/ISRCTN43900695**).

Why have I been selected to take part and what are the exclusion criteria?

We are looking for people who are over 18 years of age, who have access to the Internet and who have can understand written English. This is because the study treatment will be delivered online and because the study questions are in English.

We are looking for people who have had a problem sleeping recently (within the last two weeks to three months) and people who are **good** sleepers (i.e. people who do not report having problems with their sleep).

If you are a poor sleeper, you would need to experience the following:

1) You have problems falling asleep, staying asleep, or waking up too early. This needs to happen for at least three nights per week. This also needs to have happened for at least two weeks, but not more than three months.
2) You are distressed by your poor sleep, or you have noticed being affected during the day by your poor sleep.

Both of these things must have happened even though you have had the opportunity for sleep.

If you are a good sleeper, you do not have any problems with your sleep.

You cannot take part if you have had a head injury, have schizophrenia, epilepsy or a personality disorder, because this might affect how well the treatment works. There are no other exclusion criteria.

Do I have to take part?
You do not have to take part in this study. If you participate, you can change your mind and withdraw from the study at any point without having to give a reason.

What will happen if I take part?
This study will be conducted entirely online and you will not need to visit Northumbria University at any point. You will be sent automatic email reminders at each point of the study.

This study is a type of study called a randomised controlled trial. This means that some people who take part will receive the treatment and some people will not receive the treatment. In addition, some people who receive the treatment will do so after a delay.

The treatment is an online version of a leaflet which we have previously used in face-to-face studies with people who have clinical sleep problems. This involves being told about various different practical behaviours which you can do to help improve your sleep. This leaflet has been shown to be effective and we want to see if the online version is also effective, because this will allow us to deliver this very cheaply and reach lots of people at once. You will be free to download, print and keep this leaflet and you will be encouraged to refer to this as often as you would like to.

The first part of the study involves completing some questionnaires about your sleep habits and sleep quality, stress levels and general mood. You will also be asked some questions in relation to the ongoing COVID-19 pandemic, and brief questions about your health and your employment. This would take approximately 30 to 45 minutes to complete.

We will then ask you to keep a daily log of your sleep for one week, by completing what is known as a 'sleep diary'. This takes about five minutes to complete each day and involves noting down details including what time you went to sleep, how long you slept for, and whether you woke up during the night or not. This will be done using an online link (which we will send to you by email) which you can complete on your computer, tablet or mobile phone. The next step of the study will depend on which group you are in.

Poor sleepers
If you are a poor sleeper, you will be randomly placed into one or two groups. If you are in the first group, you will receive the treatment leaflet after keeping your sleep diary for a week.

If you are in the second group, after completing the assessments, you will be asked to wait approximately one month. We will then send you an email which will ask you to repeat some of the questionnaires that you were asked to complete at the start of the study (which will only take a few minutes) and you will then be asked to keep a sleep diary for a week before being given the treatment leaflet.

This delay means that we can measure if the treatment works but still make sure that everyone who might benefit from the treatment receives it.

All poor sleepers will be asked to keep sleep diaries for another week after receiving the treatment leaflet. At the end of this week, you will be asked to complete some of the same questions you completed at the start of the study. This means that we can check how effective the treatment leaflet is in the short-term.

You will also be asked to repeat these questions one month and three months after receiving the treatment leaflet. This allows us to see if the treatment is effective over a longer time period.

Good sleepers
If you are a good sleeper, you will be randomly placed into one of two groups. If you are in the first group, you will complete sleep diaries for one week before receiving the treatment leaflet. You will then be asked to complete sleep diaries for another week. At the end of each week, you will be asked to complete some of the same questions

you completed at the start of the study. This means that we can check how effective the treatment leaflet is in the short-term. You will also be asked to repeat these questions one month and three months after receiving the treatment. This allows us to see if the treatment is effective over a longer time period.

If you are in the second group, you will not receive the treatment leaflet and will instead keep sleep diaries for two weeks. At the end of each week, you will be asked to complete some of the same questions you completed at the start of the study. You will also be asked to repeat these questions one month and three months after this point.

The reason we do this is because we are interested in tracking the sleep of good sleepers, who do not report having any problems with their sleep, throughout the COVID-19 pandemic. This is because doing this may help us to develop new behavioural treatments for poor sleep.

What are the possible advantages or disadvantages of taking part?

By taking part in this study, you will be helping to improve potential treatments for sleep problems.

In terms of direct advantages, the potential advantages of taking part in this study are that you may receive treatment which might help your current sleep problems. This treatment may also prevent future sleep problems from developing. If you do not receive the treatment, you will not receive any direct advantage but you will still be helping to improve treatments for sleep problems. Many people report a feeling of satisfaction from aiding research that will help other people in the future.

There are very unlikely to be any disadvantages of taking part in this study. We have used this treatment in the past, in multiple face-to-face research studies with people who have long-term sleep problems, and there have not been any side effects.

If you are concerned about the potential psychological impact of taking part, you should contact your GP. If you are concerned about your physical health, or need further information with regarding COVID-19, you should contact your GP, or refer to the NHS 111 website (www.111.nhs.uk) for further information.

There are no financial rewards for participation.

How will confidentiality be assured?

We take confidentiality very seriously.

We will ask you for some potentially identifiable information, such as your ethnic background, occupation and postcode. This will not be examined at an individual level. This information is needed because we want to understand if particular groups of people might be at risk of developing sleep problems within the context of this pandemic (e.g. healthcare workers, or people with certain health conditions, or in people who have recently been made unemployed) and if the treatment could be

targeted towards these groups of people. You do not have to provide this information if you do not wish to, and we will not ask you for your name.

You will be asked to provide an email address so that we can send you a link to the study and a daily reminder to complete your sleep diary. Your email address will also be used to provide you with a link to the treatment leaflet, and to send you reminders at each follow-up stage of the study (one week, one month and three months). Your email address will not be used for any other purpose and will be deleted at the end of the study.

We will ask you to create a code word so that if you need to withdraw your information at any point, we will be able to do this.

How will my data be stored and who will have access to the information that I provide?

If you take part in this study, any information which you provide will be treated confidentially. This information will be stored securely in password-protected files on Northumbria University computer storage and will only be accessible by authorised members of research team. With your permission, we will retain the data in anonymised form indefinitely for use in the future and similar studies. All data will be stored in accordance with Northumbria University Guidelines and in accordance with General Data Protection Regulations (GDPR).

Should the research be presented or published in any form, all data will be anonymous (i.e. your personal information or data will not be identifiable). This anonymous data may be held indefinitely to ensure research integrity.

Any personally identifiable information and data gathered during this research is subject to and will be stored in line with EU General Data Protection Regulation (GDPR) and the UK Data Protection Act (2018). Any personally identifiable information will be destroyed as soon as it is no longer needed (e.g. email addresses used to keep in contact with you will be deleted at the end of the study). Any IP addresses collected via online survey systems will be deleted as soon as data collection is complete.

If personal data has been collected during this study, the legal basis for the study's personal data processing is that the research is being conducted in the public interest, and/or is necessary for scientific and historical research purposes. You have the right to access your data upon request. Contact the Information Commissioner's Office for further information, and/or complaints about the University's processing of personal data: https://ico.org.uk/. The Data Protection Officer at Northumbria University (the Data Controller) is Duncan James (dp.officer@northumbria.ac.uk)

What will happen to the results of the study?

The overall results of this study will be used in scientific publications and conference presentations.

We will also seek to make the results of the study, in summary form, available elsewhere (e.g. the Northumbria University website) and the results will also be

available upon request, by contacting the research team. The results will also be available on this webpage (https://www.isrctn.com/ISRCTN43900695).

Who has reviewed this study?

This study has been approved by the University Ethical Approval system (Ref: 23377) at Northumbria University.

Contact for further information:

Dr. Greg Elder

Northumbria Sleep Research Laboratory

Northumbria University

Newcastle upon Tyne

NE1 8ST

United Kingdom

Email: g.elder@northumbria.ac.uk

If you have any concerns regarding this research or if you wish to register a complaint, please direct it to the Department of Psychology Ethics Chair at nick.neave@northumbria.ac.uk

This study and its protocol have received full ethical approval from the Department of Psychology Ethics Committee in accordance with the School of Health and Life Sciences Ethics Committee. If you require confirmation of this please contact the Chair of this Committee (Professor Nick Neave), stating the title of the research project and the name of the researcher.

Please note that Northumbria staff who participate in this study are expected to do so in their own time.

23. **APPENDIX 2: ONLINE CONSENT FORM**

If you would like to take part in this study, please read the statement below and click 'I agree'.

By clicking on "I agree", I indicate that I understand the nature of the study, and what is required from me. I understand that after I participate I will receive a debrief providing me with information about the study and contact details for the researcher.

I understand I am free to withdraw from the study at any time, without having to give a reason for withdrawing. I agree to provide information to the investigator and understand that my contribution will remain confidential.

I agree **I disagree**

I consent for my data to be used in anonymised form in similar, national and international studies.

I agree **I disagree**

Thank you for agreeing to take part in this study. Before you start the study we would like you to provide us with an anonymous (but memorable) code word in the box below. This code word will be used to identify your data should you want to withdraw from the study at a later date. Please choose a memorable code word, but try to avoid using obvious phrases (e.g., 'code word') or phrases that may compromise the anonymity of the data (e.g., your name).

24. APPENDIX 3: INTERVENTION (SELF-HELP LEAFLET)

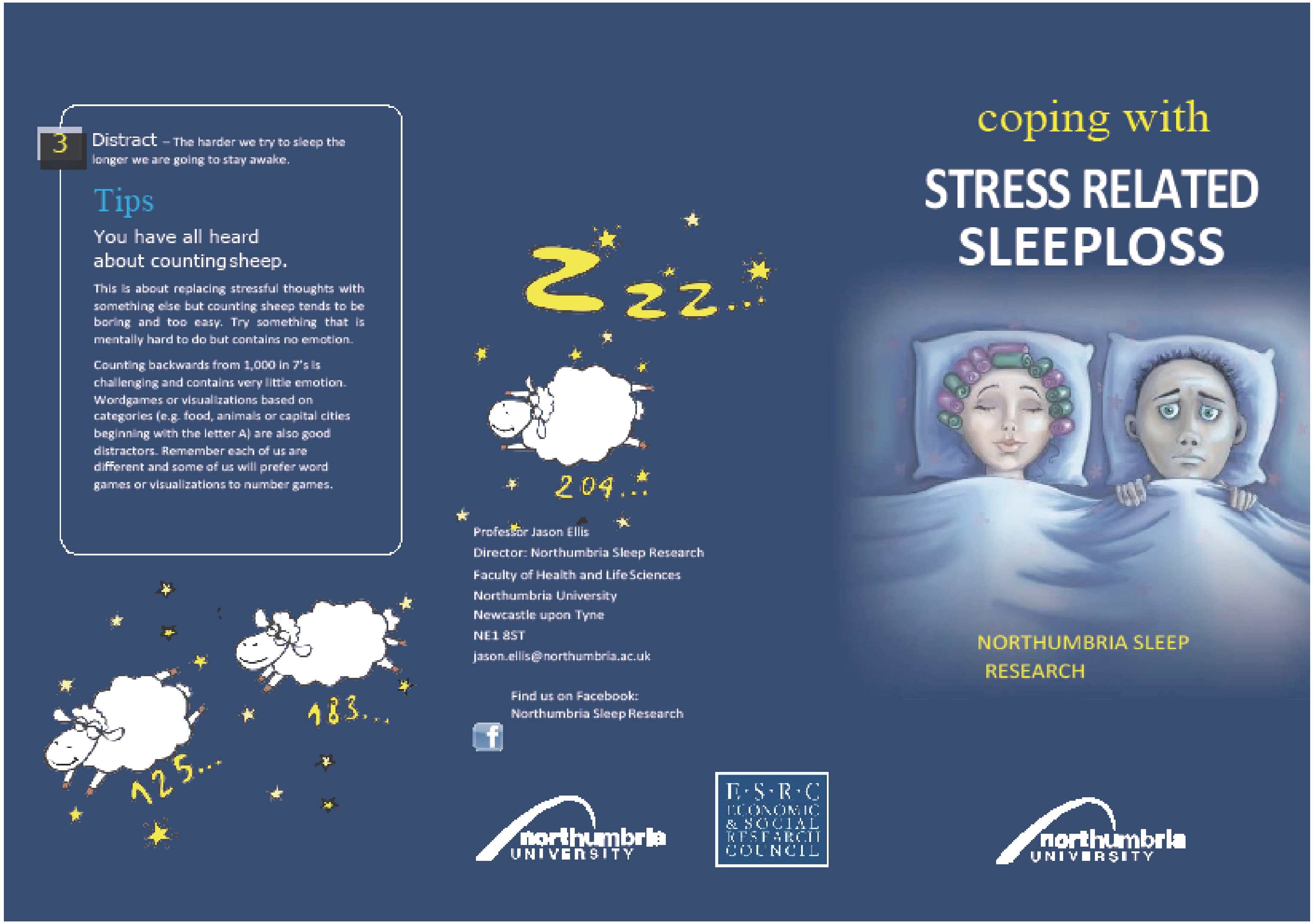

What is Short term Insomnia?

Short term Insomnia is a condition where an individual has a difficulty in getting off to sleep, or staying asleep, or naturally awakening too early in the morning, despite plenty of opportunity to sleep. These problems occur three or more nights in any given week and this problem should have been going on for anywhere between two-weeks and three months. In most cases short term Insomnia will start following a major life event (such as an illness or stressful experience) but as the stress or illness gets under control the insomnia remains.

The first thing to remember is that insomnia, in its acute or short-term form, is a normal biological reaction to stress. In most cases your sleep should return back to normal either when the stress goes away or you begin to cope with the stress.

Is it a problem with my bedroom?

It is unlikely that your bedroom environment is the root cause of your insomnia but it can make your symptoms worse and in some cases maintain the vicious cycle of insomnia. To that end, a healthy bedroom environment is one that is cool, dark, and quiet.

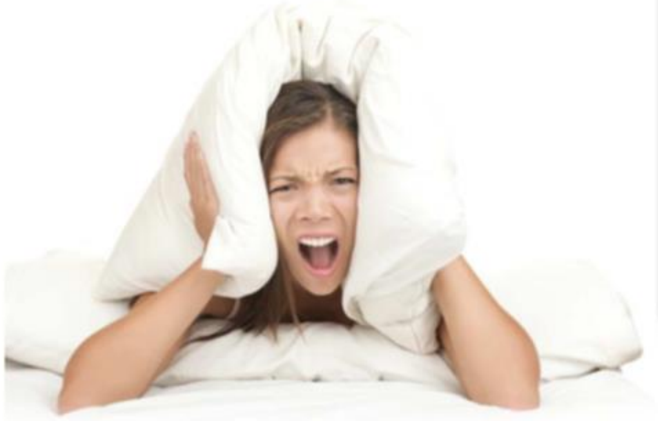

Dealing with my short term Insomnia.

There are three simple rules to help you deal with your short term Insomnia (the three D's) and help prevent it from becoming a chronic problem:

1 Detect - It is important to determine if the problem is short term Insomnia, if it is another sleep disorder, or if it is being caused by something or someone else...

Tips

Keep a sleep diary.

Make a note of what time you go to bed, how long it takes you to get off to sleep, if you wake in the night, what times and for how long, and what time you get up. This information can help identify any patterns in your sleep. As short term Insomnia tends to be quite unpredictable, a pattern of awakening at the same time every night may indicate problem other than insomnia.

Talk to your GP.

The sooner you discuss insomnia with your GP (with your sleep diary) the more likely you will get help and determine if there is a physical problem. What causes insomnia is rarely the same thing that maintains it over the longer-term so getting it sorted early can prevent it from becoming a chronic problem. If you are on any medications, talk to your GP about the type, dose, and timing of your medication. See if there is a chance that your meds are interfering with your sleep or if any changes in regimen might resolve your insomnia.

2 Detach - The longer we spend in bed awake the more likely we get anxious, frustrated, and angry (which is not the best combination for sleeping).

Tips

Use the bedroom for sleep or sex alone.

If you are in bed, not asleep, and find yourself getting anxious or frustrated, leave the bedroom and return when you feel sleepy. What to do in the mean time? Reading is a great idea although a magazine or newspaper is preferable to a book as magazine articles tend to be short whereas you can easily get caught up in a book and fight the sleepiness. Conversely, only sleep in bed. Sleeping in other environments, such as on the sofa, weakens the association between the bed and sleep and makes it harder to sleep in your bed when you do go to sleep.

Put the day to bed before you go to bed.

Make a plan to wind down, at least an hour or two before bedtime. Put the laptop down, close down your email, and set aside some time to do something relaxing. A perfect way to break up the day from the night is to complete a daily diary of what you have achieved today, what you have to do tomorrow, and what things you have or need to put into place to deal with tomorrows challenges. Not only does this put the day to rest but also clears out the mind and gives you a sense of control.

25. APPENDIX 4: PARTICIPANT DEBRIEF SHEET

Project Title: Testing an early online intervention for the treatment of disturbed sleep during the COVID-19 pandemic (Sleep COVID-19)

What was the purpose of the project?

Thank you for taking part in this study.

Stressful life events can cause short-term periods of sleep disruption, and the ongoing COVID-19 (coronavirus) pandemic is likely to represent one such stressful life event. Sometimes short-term sleep disruptions can lead to insomnia, which is where the sleep disruption becomes a long-term problem. Sometimes this is due to behaviours such as spending too much time in bed, or worrying about sleep during the next day. We wanted to trial a treatment to stop sleep disruption, occurring as a result of the stress associated with the ongoing COVID-19 pandemic, becoming a problem.

The aim of this study was to examine whether a brief online treatment, which tries to stop people from engaging in those behaviours, can help sleep problems in the short term (one week after receiving it). We also wanted to know if it could help sleep problems in the long-term (one and three months after receiving the treatment). We also wanted to know what the effects were in people without sleep problems, because we want to see if we can use this treatment as an early intervention to stop people developing sleep problems in the first place.

This online treatment leaflet was based on an information leaflet which we have previously used with people who have long-term sleep problems (insomnia). We have previously found that this has been effective in reducing insomnia symptoms in people with short-term sleep disruptions. We expect to find that the online treatment is effective in this study in people with poor sleep. We will be able to use the results of this study to design more effective interventions for people who have short-term sleep disruption.

If you are concerned about the potential psychological impact of taking part, you should contact your GP. If you are concerned about your physical health, or need further information with regarding COVID-19, you should contact your GP, or refer to the NHS 111 website (www.111.nhs.uk) for further information.

How will I find out about the results?

You will be able to find out the results by visiting the following internet page (https://www.isrctn.com/ISRCTN43900695) We expect that the study will be completed by early 2022.

You will also be able to obtain a summary of the results by emailing the principal investigator (Dr Greg Elder: g.elder@northumbria.ac.uk). We may also place a summary of the results on a web page on the Northumbria University website.
**Have I been deceived in any way during the project?**<br><br>No. You have not been deceived in any way during this study.

**If I change my mind and wish to withdraw the information I have provided, how do I do this?**<br><br>Please contact the principal investigator (Dr Greg Elder: g.elder@northumbria.ac.uk) by email, with your code word. Please do this within one week of participation as it will not be possible to remove and delete your data after this point.

If you have any concerns regarding this research or if you wish to register a complaint, please direct it to the Department of Psychology Ethics Chair at nick.neave@northumbria.ac.uk

This study and its protocol have received full ethical approval from the Department of Psychology Ethics Committee in accordance with the School of Health and Life Sciences Ethics Committee. If you require confirmation of this please contact the Chair of this Committee (Professor Nick Neave), stating the title of the research project and the name of the researcher.

The data collected in this study may also be published in scientific journals or presented at conferences. Information and data gathered during this research study will only be available to the research team named above, and the Chair of Ethics (Professor Nick Neave). Should the research be presented or published in any form, all data will be anonymous (i.e. your personal information or data will not be identifiable). This anonymous data may be held indefinitely to ensure research integrity.

Any personally identifiable information and data gathered during this research is subject to and will be stored in line with EU General Data Protection Regulation (GDPR) and the UK Data Protection Act (2018). Any personally identifiable information will be destroyed as soon as it is no longer needed (e.g. email addresses used to keep in contact with you will be destroyed as soon as they are no longer required). Any IP addresses collected via online survey systems will be deleted as soon as data collection is complete.

If personal data has been collected during this study, the legal basis for the study's personal data processing is that the research is being conducted in the public interest, and/or is necessary for scientific and historical research purposes. You have the right to access your data upon request. Contact the Information Commissioner's Office for further information, and/or complaints about the University's processing of personal data: https://ico.org.uk/. The Data Protection Officer at Northumbria University (the Data Controller) is Duncan James (dp.officer@northumbria.ac.uk)

26. APPENDIX 5: DEMOGRAPHIC AND HEALTH QUESTIONS

How many children (under 18) live in your household? ▼ 0 (4) ... 10 or more (14)

Have you been formally diagnosed with COVID-19? Yes (1) / No (2)

Have you displayed symptoms of COVID-19? Yes (1) / No (2)

Have you received a test for COVID-19? Yes (1) / No (2)

Which country do you currently live in? UK (1) / USA (2) / Other (please specify) (3)

- For UK respondents only: What is your postcode? Please type your answer.
- For USA respondents only: Which state do you live in? Please type your answer.

Are you currently (please select as many boxes as apply):
In active paid work (1) / Unemployed and seeking work (2) / Retired (3) / Unemployed due to illness or disability (4) / Doing voluntary work (5) / At home doing housework (6) / A full-time student (7) / Other (please specify) (8)

What is your occupation? Please complete this for your current, or your most recent, paid job (free text)

Is / was this job full-time or part-time? Full-time (1) / Part-time (2)

Please provide a brief description of your job role (what does your job involve?) (free text)

Please state approximately how many hours per week you spent doing this job ▼ 0-5 (8) ... 51+ (18)

Are/were you an: Employee (1) / Employer (2) / Self-employed (3) / Supervisor/Foreman (4)

How many people do/did you supervise?

Have you been placed on furlough (temporary leave) by your employer at any point as a result of the COVID-19 pandemic? Yes (1) / No (2)

Have you became unemployed, or are you being made redundant, at any point as a result of the COVID-19 pandemic? Yes (1) / No (2)

Are you now working at home as a direct result of the COVID-19 pandemic? Yes (1) / No (2)

What is your month of birth?

What year were you born?

What is your gender? Male (1) / Female (2) / Other - please specify (3)

If you are female, would you consider yourself to be: Pre-menopausal (1) / Experiencing the menopause now (2) / Post-menopausal (3)

Which of the following best describes your ethnic background?
Please tick one of the following groups. If applicable, please specify your ethnic origin. White (1) / Black - Caribbean (2) / Black - African (3) / Black - Other (4) / Pakistani (5) / Bangladeshi (6) / Chinese (7) / Asian - Other (8) / Other - please specify (9)

Which of the following best describes your marital status? Please tick one of the following: Single (1) / Married (2) / Widowed (3) / Divorced (4) / Separated (5) / Living as married (6) / Other (please specify) (7)

Do you know your approximate height in feet and inches, or centimetres? Feet and inches (1) / Centimetres (2)

- Please provide your approximate height (feet) & Please provide your approximate height (inches) **OR**
- Please provide your approximate height (cm)

Do you know your approximate weight in stone and pounds, or kilograms (kg)? stone and pounds (1) / kilograms (2)

- Please estimate your weight (stone) & Please estimate your weight (pounds) **OR**
- **p**lease estimate your weight (in kilograms)

What type of accommodation do you normally live in? Owner-occupier (1) / Privately rented (2) / Council/housing association rented (3) / Residential home (4) / Nursing home (5) / Sheltered / accommodation (6) / Other (please specify) (7)

How many adults live in your household?

Do you have any of these qualifications? Please tick all that apply. CSE / O levels / School Certificate / GCSE (1) / City and Guilds Certificate (2) / A levels / Highers / BTEC (3) / Recognised Trade Apprenticeship (4) / HND (5) / Clerical / Commercial Qualification (6) / First degree (BA, BSc etc) (7) / Higher Degree (MSc, PhD etc) (8) / Medical / Nursing / Teaching qualifications (9) / Membership of Professional Institute (10) / Other (Please describe) (11)

Do you currently have a physical or psychiatric illness? Yes (1) / No (2) Please provide further details. (if applicable)

How many days per week do you consume alcohol?

On days when you have anything alcoholic to drink, how many units do you normally have? (Half-pint normal strength beer =1 unit, small glass wine= 1.5 units) ▼ 0-5 (4) ... 25+ (9)

Do you currently take any medication? Yes (1) / No (2)

(if applicable) Please list the medication which you are currently taking (i.e. which you have been put on in the past 3 months): Medication name or tradename (1) / dosage (2) / frequency (3)

27. APPENDIX 6: EXAMPLE SOCIAL MEDIA ADVERTISEMENT

Are you experiencing poor sleep during the ongoing COVID-19 (coronavirus) pandemic? Northumbria Sleep Research are running an online treatment study for poor sleepers. This treatment is based on our work with people who have insomnia and aims to help improve your sleep by changing your behaviour. There is no charge for the treatment.

We are also looking for good sleepers who will also receive the treatment, and good sleepers who are willing to have their sleep monitored using online sleep diaries so that we can track the impact upon sleep over time.

For further details please email g.elder@northumbria.ac.uk

This study has been approved by the University Ethical Approval system (Ref: 23377) at Northumbria University.
